# Case Report: Exacerbation of varices following atezolizumab plus bevacizumab treatment of hepatocellular carcinoma: A case series and literature review

**DOI:** 10.3389/fonc.2022.948293

**Published:** 2022-07-22

**Authors:** Hiroyuki Suzuki, Hideki Iwamoto, Shigeo Shimose, Takashi Niizeki, Tomotake Shirono, Yu Noda, Naoki Kamachi, Taizo Yamaguchi, Masahito Nakano, Ryoko Kuromatsu, Hironori Koga, Takumi Kawaguchi

**Affiliations:** ^1^ Division of Gastroenterology, Department of Medicine, Kurume University School of Medicine, Kurume, Japan; ^2^ Iwamoto Internal Medicine Clinic, Kitakyushu, Japan

**Keywords:** atezolizumab, bevacizumab, hepatocellular carcinoma, gastrointestinal hemorrhage, portal hypertension, varices

## Abstract

Recently, a combined regimen of atezolizumab and bevacizumab (AB) treatment has been approved as a first-line treatment in patients with advanced hepatocellular carcinoma (HCC), contributing to prolonged survival. However, we often encounter cases where treatment must be discontinued due to the occurrence of adverse events. One of these events, which is often fatal, is gastrointestinal bleeding. To clarify the clinical effects of gastrointestinal bleeding after AB treatment, we evaluated patients with HCC who were treated with AB at our institution. Of the 105 patients, five treated with AB developed gastrointestinal bleeding, necessitating treatment discontinuation. Additionally, we encountered two cases where exacerbation of varicose veins was observed, and AB therapy could be continued by preventive treatment of varices. In conclusion, an appropriate follow-up is required during treatment with AB to prevent possible exacerbation of varicose veins.

## Introduction

Hepatocellular carcinoma (HCC) is the most frequently occurring primary liver malignancy and a major cause of cancer-related deaths worldwide (1). It typically occurs in patients with persistent hepatitis or cirrhosis secondary to hepatitis B or C virus infection, excessive alcohol consumption, and nonalcoholic steatohepatitis (2, 3). Although advances in diagnosis have contributed to a reduced risk of developing HCC, this malignancy is often diagnosed in advanced stages (4). Recently, systemic therapy for advanced HCC has achieved remarkable progress, and various molecular targeted agents (MTAs), primarily inhibiting vascular endothelial growth factor (VEGF) signaling, have been approved (3). In the past few years, immune checkpoint inhibitors (ICIs), which target tumor immunity, have caused a paradigm shift in the treatment of advanced HCC (3). Patients with HCC having liver cirrhosis, particularly decompensated liver cirrhosis, often experience complications, such as varicose veins, associated with portal hypertension, hepatic encephalopathy, and hepatorenal syndrome (5, 6). Treatment of advanced stages of HCC involving MTAs and ICIs, often aggravates these complications, rendering treatment continuation difficult (3). Therefore, a comprehensive follow-up prevents the exacerbation of these complications is required.

A combination of atezolizumab and bevacizumab (AB) has been recently approved as a first-line treatment in patients with advanced HCC, as it shows superiority to sorafenib in terms of survival benefits (7). However, information regarding potential predictors of response, resistance, and toxicity of immunotherapy for HCC is very limited, therefore, clarifying these indicators is of pivotal importance when using this combination therapy in a clinical setting (8–10). Even after excluding patients at high risk for bleeding, approximately 10% of the patients in the study experienced bleeding during AB treatment, and 6%–10% of them developed > grade 3 hemorrhage (7, 11). In such circumstances, HCC treatment has to be discontinued to stop the bleeding, favoring HCC progression. Therefore, elucidating the clinical features of gastrointestinal bleeding due to AB treatment is imperative.

Herein, we present a case series and literature review of exacerbated varices and/or gastrointestinal bleeding during AB combination treatment.

## Materials and methods

In this study, we evaluated 105 patients with advanced HCC who received AB treatment between January 2020 and December 2021 at our institute (Kurume University Hospital and Iwamoto Internal Medicine Clinic). Endoscopic evaluation was not performed in cases where collateral blood vessels were not found on computed tomography or other modalities or when patients had chronic hepatitis instead of cirrhosis, as indicated by clinical evaluation. Out of 105 patients, 85 of them underwent endoscopic evaluation for varices prior to AB treatment. To assess splenomegaly, we calculated the splenic index based on the formula (craniocaudal dimension × width × thickness) and defined patients with splenomegaly as having a splenic index > 480 (12).

## Case presentation

**Figure 1:**
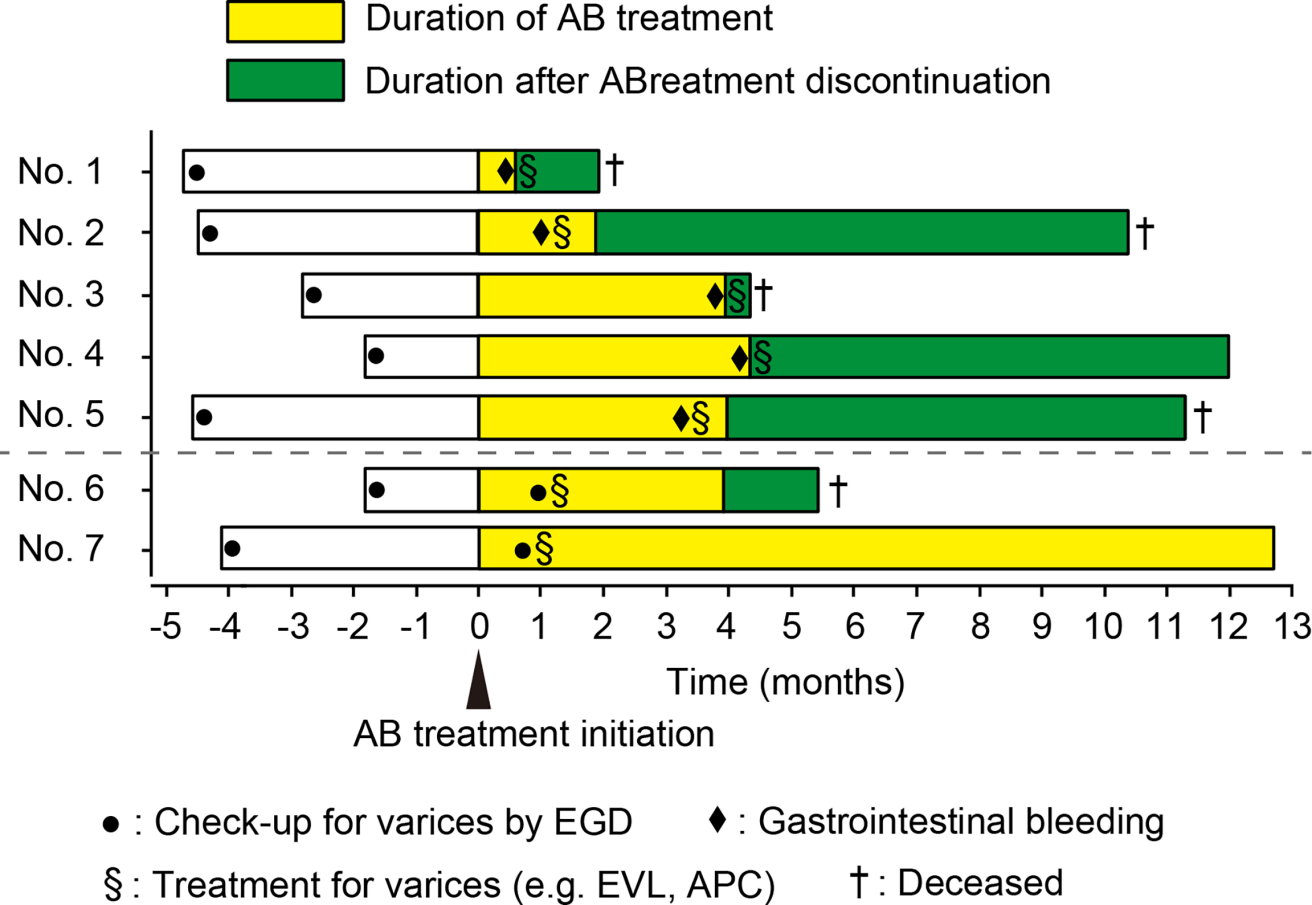
Swimmer plot for all cases. AB, atezolizumab plus bevacizumab; EVL, endoscopic variceal ligation; APC, argon plasma coagulation. EGD, esophagogastroduodenoscopy.

Of the 85 patients who underwent endoscopic evaluation prior to AB treatment, 12 had form 1 of varicose veins. In 55 patients, follow-up endoscopy could not be performed after initiation of the treatment due to deterioration of general condition, progression of HCC, or lack of scheduled endoscopic follow-up. Thirty patients underwent follow-up endoscopy after AB treatment. Out of the 105 patients, 4.8% (5/105) developed gastrointestinal bleeding as an adverse event (AE) and 1.9% (2/105) had rapid exacerbated varices during AB therapy. Patient characteristics at baseline are summarized in **Table 1**. The clinical course of patients is shown in **Figure 1**.

**Table 1:** Baseline patient characteristics for current report and previously reported cases.

Case	Age	Sex	Child-Pugh class, score	ALBIgrade, score	Tumor stage TNM, BCLC	History of MTAtreatment	History of hepaticresection	Etiology	Platelet(×10^5^/μl)	Splenomegaly, splenic index	Reason for discontinuation of AB treatment
1	70	M	A, 6	2, -1.81	IVB, C	–	–	HCV (SVR)	4.9	+, 985	AE
2	78	M	A, 6	2, -2.01	III, B	+	+	NASH	9.4	-, 391	AE
3	61	M	B, 7	2, -1.50	IVB, C	+	+	HCV	12.0	+, 793	AE
4	55	M	A, 5	2, -2.57	IVB, C	–	–	NASH	7.7	+, 1688	AE
5	69	M	A, 5	2, -2.45	III, B	–	+	Alcohol	7.6	+, 2099	AE
6	37	M	A, 6	2, -1.67	IVB, C	+	+	NASH	7.7	+, 553	PD
7	72	M	A, 6	2, -1.94	III, B	–	–	Alcohol	6.2	+, 1136	–
Median (range)	69 (37–78)			-1.94 (-1.67–2.57)					7.7 (4.9–12.0)	985 (391–2099)	
13, Case 1	60s	M	A, 5	2, -2.78	IVB, C	+	+	HCV (SVR)	11.1	Spleen volume, 193 cm^3^	NA
13, Case 2	60s	M	A, 6	2, -2.16	III, B	+	–	HCV (SVR)	12.6	Spleen volume, 262 cm^3^	NA

ALBI, albumin-bilirubin index; BCLC, Barcelona Clinic Liver Cancer; MTA, molecular targeted agent; AB, atezolizumab plus bevacizumab; HCV, hepatitis C virus; SVR, sustained virological response; AE, adverse event; NASH, non-alcoholic steatohepatitis; PD, progressive disease; Ref., reference; NA, not available.

### Five patients developed gastrointestinal bleeding during AB treatment

In the following five cases, bleeding was observed 76.8 (range: 18–132) days after initiation of AB treatment.

#### Case 1

**Figure 2:**
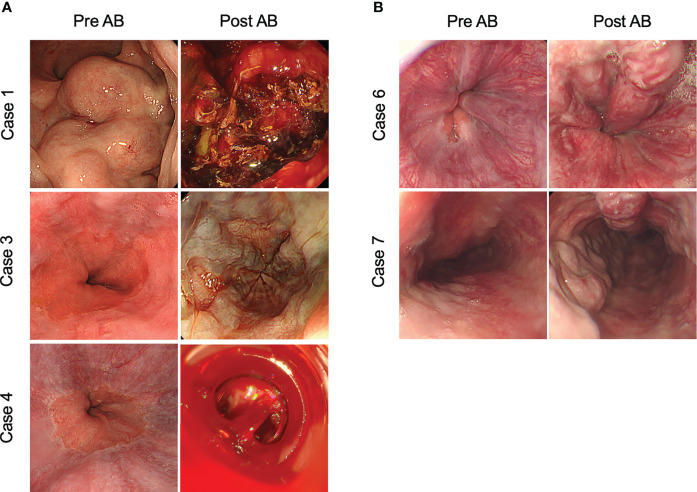
Representative images of gastrointestinal bleeding and exacerbation of varicose veins after atezolizumab plus bevacizumab treatment. **(A)** Patients that developed gastrointestinal bleeding after treatment with atezolizumab plus bevacizumab. **(B)** Patients in which varices exacerbated during atezolizumab plus bevacizumab treatment. AB, atezolizumab plus bevacizumab treatment.

A 70-year-old Japanese man with stage IVB HCC with lung metastasis was treated with AB. He had splenomegaly and developed collateral circulation in the inferior vena cava. Seventeen days after treatment initiation, a large blood volume was observed in the patient’s stool. Colonoscopy revealed hemorrhage from the rectal varicose vein, which was detected as grade III form of varices, and the red color sign was negative before treatment (**Figure 2A**). Immediate endoscopic treatment, including endoscopic variceal ligation (EVL) with endoscopic injection sclerotherapy, was performed. However, the hemostatic effect was poor, leading to cardiopulmonary arrest. Bleeding continued, and the patient eventually died 39 days after the occurrence of the first bleeding event.

#### Case 2

A 78-year-old Japanese man with unresectable HCC (stage III) was treated with AB. Two weeks after treatment initiation, the patient developed melena and grade 3 liver dysfunction. Esophagogastroduodenoscopy (EGD) revealed bleeding from the esophageal varices, detected as Li F1 Cb RC(-) before treatment. EVL was performed immediately, and hemostasis was achieved. Although endoscopic findings 1 month after varicose vein treatment indicated only esophageal varicose vein [Li F1 Cb RC(-)], the AB therapy was discontinued due to the event of bleeding from esophageal varices. Eventually, due to HCC progression, the patient died 9 months after the varicose vein ruptured.

#### Case 3

A 61-year-old Japanese man with stage IVB HCC with lung metastasis was treated with AB. After three cycles of AB treatment, computed tomography demonstrated stable disease condition according to the Response Evaluation Criteria in Solid Tumors (RECIST) version 1.1. Four cycles after treatment initiation, the patient experienced hematemesis and was admitted to our hospital. EGD revealed bleeding from esophageal varices, detected as Lm F1 Cb RC(-) before treatment. EVL was immediately performed, and temporary hemostasis was achieved. However, his liver function and general condition rapidly deteriorated after the varix rupture, and then the patient died 10 days after the event. (**Figure 2A**).

#### Case 4

A 55-year-old Japanese man with stage IVB HCC with bone metastasis was treated with AB. Four months after treatment initiation, the patient experienced hematemesis and was admitted to the hospital. EGD revealed hemorrhage from the esophageal varices, detected as Li F1 Cb RC(-) before treatment (**Figure 2A**). EVL was immediately performed, and hemostasis was achieved; however, AB treatment was discontinued. Subsequent endoscopy showed no apparent exacerbation of varicose veins. The patient is alive and is being treated with percutaneous radiofrequency ablation for HCC.

#### Case 5

A 69-year-old Japanese man with unresectable HCC (stage III) was treated with AB. After three courses of AB treatment, computed tomography demonstrated a partial response to treatment, according to the RECIST ver.1.1. Five cycles after treatment initiation, the patient presented with hematemesis and was admitted to our hospital. EGD revealed bleeding from the esophageal varices, detected as Li F1 Cb RC(-) before treatment. EVL was immediately performed, and hemostasis was achieved. Endoscopic findings 1 month after varicose vein treatment showed only esophageal varices [Li F1 Cb RC(-)]. However, the AB therapy was discontinued due to the event of bleeding from esophageal varices. Thereafter, atezolizumab monotherapy was initiated in accordance with the patient’s wishes. Eventually, due to HCC progression, the patient died 7 months after the varicose vein ruptured.

### Two cases of exacerbated varices during AB treatment

#### Case 6

A 37-year-old Japanese man with stage IVB HCC with peritoneal dissemination was treated with AB combination therapy. EGD performed 4 weeks after treatment revealed worsened esophageal varices [Li F3 Cb RC(-)], detected as Li F1 Cb RC(-) before AB treatment initiation (**Figure 2B**). Thereafter, EVL was performed to prevent bleeding. Due to the progression of HCC, the patient died 5 months after the initiation of combined AB treatment.

#### Case 7

A 72-year-old Japanese man with unresectable HCC (stage III) received AB treatment. EGD performed 3 weeks after treatment initiation revealed rapidly exacerbated esophageal varices [Li F3 Cb RC(-)], detected as Li F1 Cb RC(-) prior to the initiation of AB treatment (**Figure 2B**). EVL and argon plasma coagulation were performed to prevent bleeding. After three courses of combination treatment, enhanced computed tomography demonstrated a partial response to treatment according to the RECIST ver.1.1. Subsequently, the combination therapy with AB was continued for more than 12 months.

## Discussion

In this study, we described the clinical features of gastrointestinal bleeding during AB treatment in patients with advanced HCC. Five patients experienced gastrointestinal bleeding. The prevalence of gastrointestinal bleeding in the current study (4.8%) was similar to that in a previous clinical trial (7). To the best of our knowledge, two cases of rapid growth of esophageal varices following AB have been reported (13). **Table 1** shows the characteristics of those patients and that of the patients in the present study. It has been reported that once gastrointestinal bleeding occurs, combination treatment continuation will be difficult in most cases for the following reasons: rapid deterioration in liver function, rapid growth of HCC, and acquired resistance against AB treatment (11, 14). Similarly, all patients in our study who developed gastrointestinal bleeding could not continue AB treatment and experienced HCC progression after treatment discontinuation. However, two patients in whom preventive treatment for varicose veins was possible, AB treatment could be continued for a longer period. Therefore, to continue treatment without gastrointestinal bleeding occurrence, appropriate follow-up and intervention for varicose veins at the right time are of utmost importance. Elective treatment modalities for non-bleeding varices, such as EVL and APC, are invasive. Therefore, it is important to consider the delayed wound healing effect of bevacizumab, which could induce varicose vein rupture after the treatment. According to the National Comprehensive Cancer Network Clinical Practice Guidelines in Oncology ver. 2.2022 (15), 4–6 weeks of intermission from the last administration of bevacizumab is recommended for surgical treatment. Therefore, if possible, we recommend that elective treatments should be performed 4–6 weeks after the last administration of bevacizumab for non-urgent cases.

It has been shown that HCC developed in the context of chronic hepatitis/cirrhosis (2, 3). Cirrhosis is closely associated with portal hypertension and has a very high risk of gastrointestinal hemorrhage due to the development of collateral circulation (16). Although the liver function of all patients in our report and a previous report (17) was well preserved (Child-Pugh score ≤ 7), splenomegaly was observed in most of the patients. It is suggested that all of them had potential portal hypertension. Consequently, it seems that there might be no association between the severity of liver cirrhosis and worsening of varicose vein or bleeding. Portal vein tumor thrombosis has been recognized to increase portal pressure, leading to exacerbation of varicose veins (17). However, portal vein tumor thrombosis was not identified in these cases.

ICIs are monoclonal antibodies that target immune checkpoint molecules (18, 19). Since these drugs directly regulate the immune system, immune checkpoint inhibitor-related adverse events (irAEs), mainly due to immune dysregulation, are often problematic in clinical practice (18, 19). Colitis and diarrhea are the most common irAEs in the gastrointestinal system. However, the frequency of irAEs in the upper gastrointestinal tract is low, the most common of which is nausea and loss of appetite (20). Thus, the association between gastrointestinal bleeding observed in this study and immune checkpoint inhibitors was considered low. As shown in the incidence rate of bleeding in the clinical trials summarized in **Table 2**, the incidence of bleeding with immune checkpoint inhibitor monotherapy is relatively low.

**Table 2:** The incidence rate of gastrointestinal bleeding in reported clinical trials of systemic chemotherapy for advanced HCC.

Trial	Line	Regimen	Gastrointestinal bleeding*	
			Any grade	Grade ≥ 3
SHARP (21)	First	Sorafenib (MTA)	NA	4%
Asia-Pacific trials of sorafenib (22)	First	Sorafenib (MTA)	2.7% (4/149)	NA
REFLECT (23)	First	Lenvatinib (MTA)	< 15%	< 15%
CheckMate 459 (24)	First	Nivolumab (ICI)	0%	0%
KEYNOTE 240 (25)	First	Pembrolizumab (ICI)	< 10%	1.1% (3/279)
IMbrave 150 (7)	First	Atezolizumab (ICI) plus bevacizumab (MTA)	7.6% (26/336)	2.9% (10/336)
RESORCE (26)	Second	Regorafenib (MTA)	NA	6% (21/374)
REACH-2 (27)	Second	Ramucirumab (MTA)	0%	0.5% (1/197)
CELESTIAL (28)	Second	Cabozantinib (MTA)	NA	0.2% (1/470)

*Gastrointestinal bleeding includes esophageal varices hemorrhage and upper gastrointestinal hemorrhage.

MTA, molecular targeted agent; ICI, immune checkpoint inhibitor; NA, not available.

Many types of MTA have been approved as systemic chemotherapy for patients with advanced HCC and have provided survival benefits (29, 30). In previous trials, it has been reported that 0%–7.6% of the patients with HCC developed bleeding during MTA treatment (**Table 2**) (7, 21–28). Most MTAs commonly exert their antitumor effects by inhibiting VEGF signaling. We have previously reported that MTAs inhibit angiogenesis *via* VEGF signaling not only in tumor micro vessels, but also in normal organs, such as the liver, pituitary gland, kidneys, and intestine, leading to the occurrence of various AEs (31). Moreover, the association between the use of anti-VEGF antibody and AEs of hemorrhage has been elucidated, and it comprises of the following: (i) poor vasodilation due to inhibition of nitric oxide, which has a vasodilatory effect, and (ii) reduction in capillary bed density (32). Inhibition of VEGF, which is responsible for the homeostasis of micro vessels, leads to a decrease in capillary bed density in the liver and an increase in pressure in the reticuloendothelial system, resulting in worsened varicose veins. To evaluate whether the amount of vascular bed in the liver was associated with varicose vein exacerbation, we looked for correlation between presence or absence of hepatectomy and varicose vein exacerbation. However, half of the patients had a history of resection with no significant findings. We also evaluated the correlation between the use of MTA for pretreatment and exacerbation of varicose veins. Nevertheless, only three patients had MTA as a history of pretreatment, and no significant findings were found.

Bevacizumab is recommended at a dose range of 5–15 mg/kg every 2–3 weeks, depending on the type of tumor and combination therapy (7, 33). For HCC, the recommended dosage of bevacizumab is 15 mg/kg after intravenous administration of 1200 mg of atezolizumab on the same day, every 3 weeks (7). At present, a method for reducing the dose of bevacizumab has not been established, and it is contraindicated to restart bevacizumab after it has caused gastrointestinal bleeding. Unfortunately, in cases where AB therapy has to be discontinued due to gastrointestinal bleeding, alternative HCC treatment strategies are limited. They include (1) atezolizumab monotherapy, as executed in Case 5 in this report, although it may have limited efficacy (7); (2) treatment with MTAs (ramucirumab, cabozantinib) since they are associated with a relatively low frequency of gastrointestinal bleeding as shown in **Table 2** **;** however, since these drugs commonly have anti-angiogenesis effect *via* VEGF signaling inhibition, the risk of gastrointestinal bleeding persists; (3) ICI plus ICI combination therapy, which is currently being evaluated in clinical trials (durvalumab plus tremelimumab, NCT03298451; ipilimumab plus nivolumab, NCT04039607), could be possible treatment options in the future. We have previously reported that in patients with advanced HCC who discontinued treatment due to AE after MTA administration, it might be possible to increase the antitumor effect while reducing the incidence of AE using the weekends-off administration method (31). Therefore, it is necessary to establish optimal methods for reducing bevacizumab dosage in cases where severe gastrointestinal bleeding could develop. In this report, gastrointestinal bleeding occurred at an average of 76.8 days after AB treatment initiation in five cases. In addition, in the two cases of varicose vein exacerbation, varicose veins worsened to the extent of requiring treatment 1 month after initiation of AB treatment. Based on these findings, it is necessary to evaluate varicose veins not only before AB treatment initiation but also early after its initiation. Regular follow-up every 3–6 months might be necessary, especially in patients at high risk for varicose vein rupture.

## Conclusion

AB treatment of advanced HCC might exacerbate varicose veins. However, appropriate prevention and treatment of varicose veins can contribute to prolonging the treatment duration.

## Data Availability Statement

The original contributions presented in the study are included in the article/supplementary material. Further inquiries can be directed to the corresponding author.

## Ethics Statement

Ethical review and approval was not required for the study on human participants in accordance with the local legislation and institutional requirements. The patients/participants provided their written informed consent to participate in this study.

## Author Contributions

HS and HI wrote the manuscript. SS, TN, TS, YN, NK, and TY participated in the acquisition of data and critical revision. RK, HK, and TK supervised the study. All authors discussed the results and contributed to the final manuscript.

## Conflict of Interest

The authors declare that the research was conducted in the absence of any commercial or financial relationships that could be construed as a potential conflict of interest.

The handling editor TK declared a shared Research Group Japanese Society of Hepatology with the author(s) at the time of review.

## Publisher’s Note

All claims expressed in this article are solely those of the authors and do not necessarily represent those of their affiliated organizations, or those of the publisher, the editors and the reviewers. Any product that may be evaluated in this article, or claim that may be made by its manufacturer, is not guaranteed or endorsed by the publisher.
